# Effects of Human-Social Capital Congruence and Environmental Dynamism on Dynamic of Encouragement and Organizational Innovation in New Ventures

**DOI:** 10.3389/fpsyg.2022.848977

**Published:** 2022-07-07

**Authors:** Yurong Lu, Wendi Cai, Xiaoliang Bi

**Affiliations:** School of Economics and Management, Tongji University, Shanghai, China

**Keywords:** capital congruence, dynamic of encouragement, environmental dynamism, organizational innovation, human capital

## Abstract

Although human capital and social capital can provide knowledge and social network for organizations, existing studies are inadequate to explore how the interaction between the two types of capital shapes organizational behaviors or organizational outcomes. The present study investigates whether the linkage of human capital to social capital was compensatory or complementary, and how they impact organizational innovation in consideration of the dynamic of encouragement. Using data from more than 200 technological new ventures in China, we analyze the associations among all the parameters through bootstrapping and response surface methods. The findings suggest that organizational innovation is stronger when human and social capital are congruent and that the dynamic of encouragement fully mediates the relationship between capital congruence and organizational innovation performance. Furthermore, environmental dynamism positively moderates the relationship between capital congruence and the dynamic of the environment, that is, the relationship is stronger for new ventures in high rather than low dynamic environments. Finally, the theoretical and managerial implications of this study are discussed.

## Introduction

Organizations are seeking development in extremely changing domestic and international environments with a high degree of uncertainty (Zahra, 1996; Sanyal and Sett, 2011; Nadkarni and Chen, 2014). The principal challenge for firms, particularly for new ventures, is how to overcome the uncertainty (Jansen et al., 2006). Prior studies have demonstrated that innovation (Freel, 2005; Chan et al., 2016) is the key to tackle uncertainty, and gain sustainable development and competitive advantage for organizations (Knights and Mccabe, 2003; Wyrwich et al., 2022). Therefore, how to motivate and maintain innovation has been the priority in a dynamic environment.

In recent years, the antecedents of innovation have become a research hotspot in the management field (Subramaniam and Youndt, 2005; Tseng et al., 2016; Bornaybarrachina et al., 2017; Salas-Vallina et al., 2020). To date, a great number of studies have explored the significance of human capital for the organization (Galperin et al., 2020; Kryscynski et al., 2021; Stern et al., 2021). Prior research indicates that human capital and social capital can provide knowledge, skill, and social network structures for organizations (Bhagavatula et al., 2010; Stam et al., 2014; Chadwick, 2017), and play a critical role in promoting innovation, thus enabling the organizations to deal with environmental challenges. Based on the resource-based view, both human and social capital, which are valuable, scarce, non-substitutable, and inimitable, are vital organizational resources (Wright and Dunford, 2001). They can be transformed into other forms of resources under the conditions of organizational engagement, motivational antecedent, and organizational capability (Liu, 2013; Bornaybarrachina et al., 2017). Yet, although previous research has shown the developmental value of human and social capital (Chisholm and Nielsen, 2009; Tasheva and Hillman, 2019; Weller et al., 2019; Wolfson and Mathieu, 2020), several important limitations remain to be addressed (Sophie and Luc, 2010; Akhavan and Hosseini, 2016). There have been two contradicting theoretical claims, compensatory or complementary, regarding the linkage between human and social capital.

One way to make sense of these contradictory findings is to consider a hitherto unexamined but important mediator of dynamic of encouragement, or an organizational ability to instill hopes among all its members. Employees are more likely to utilize knowledge and social network resources to manage difficult and uncertain tasks when considering that their actions can lead to positive results (Staw et al., 1994; Huy, 1999). This perception is particularly important, because lots of positive emotions among members are required in the integration, reconstruction, and transformation of resources. Huy (1999) emphasized that organizational groups produce a kind of emotional energy and some relevant capabilities of guidance and regulation, which, as important organizational capabilities, can either provide emotional motivation or barriers to organizational resource transformation and creative activity. However, extant research is inadequate to examine the effects of organizational emotional capability (e.g., dynamic of encouragement) on organizational consequences, particularly lacking empirical analyses in a dynamic environment (Akgün et al., 2008, 2009, 2011).

Furthermore, new ventures are typical organizations with strong innovative inclinations and resource demands. They show some obvious and unique characteristics in creative and innovative activities, like path ambiguity, time limitations, and skill specificity. These characteristics determine that the surrounding resources (such as knowledge and social network) should be organized to enhance product and technological innovation by means of developing new markets and technologies to cope with the threats resulting from uncertainty and the high risk of innovative activities (Wang and Fang, 2012; Bornaybarrachina et al., 2017), particularly the specialized resources such as knowledge, skills, and experience, as well as network resources such as cooperation, sharing, and communication (Klyver and Schenkel, 2013; Zane and Decarolis, 2016). These resources, in turn, maintain or increase strategic certainty, plan accuracy, and skill exploration (Venkatraman, 1989; Teece et al., 1997; Meyer et al., 2009; Ganter and Hecker, 2013) while meeting the organizational needs of innovative resources (Zahra, 1996). Additionally, Semrau and Hopp (2016) stated that most new ventures typically have fairly modest capital requirements. Chahine and Zhang (2020) examined the roles of Chief Executive Officer human capital and Chief Executive Officer change before the initial public offering. Thus, we focus on (a) the link between human-social capital congruence and the dynamic of encouragement, (b) the human-social capital congruence-organizational innovation relationship, (c) the mediation effect of the dynamic of encouragement, and (d) the moderation effect of environmental dynamism.

Drawing on the perspective of resource conversion, this study makes several notable contributions. First, our study contributes to capital congruence literature by demonstrating the necessity for new ventures to coordinate human and social capital. Second, we clarify how capital congruence influences organizational innovation for new ventures. A study on entrepreneurs showed that the human capital and financial social capital of nascent entrepreneurs positively interact in shaping individual outcomes, yet a negative interaction was found between the human capital and informational social capital (Semrau and Hopp, 2016). Previous research suggested that the combination of human and social capital is important in shaping outcomes (Hollenbeck and Jamieson, 2015; Semrau and Hopp, 2016; Kryscynski et al., 2021), yet its mechanism remains unclear in an organizational context. Finally, we introduce dynamic of encouragement and environmental dynamics into the mechanism of the relationship between capital congruence and organizational innovation, and build a mediation model with the dynamic of encouragement as a mediator and environmental dynamism as the moderator. This study sheds light on the intrinsic mechanism of capital congruence on organizational innovation from the perspective of resources.

## Theory and Hypothesis Development

### Human-Social Capital Congruence and Dynamic of Encouragement

Intellectual capital is regarded as a resource of organizational knowledge and the collection of all the knowledge in organizations by prior studies (Youndt and Snell, 2004; Youndt et al., 2004). Subramaniam and Youndt (2005) suggested that human capital and social capital are two primary aspects of intellectual capital, and there is a significant synergistic effect between them (Mosey and Wright, 2007; Wolfson and Mathieu, 2020). Specifically, the diverse thoughts and ideas resulting from human capital enable firms to facilitate social capital, and the potential of social capital, in turn, can link these ideas to make unusual and unpredictable combinations, and thus intensify the benefits of human capital (Hollenbeck and Jamieson, 2015). In spite of this argument, there are two contradictory claims, namely, congruence and in-congruence, regarding human and social capital combinations.

At present, research on the human-social capital interaction in new ventures presents multiple perspectives, particularly the access perspective and the utilization perspective (Klyver and Schenkel, 2013; Semrau and Hopp, 2016). On one hand, the access perspective argues that human capital may be helpful for the formation of network relationships and access to social capital, and that human capital is the basis for the formation of network relations in social capital. Considerable empirical evidence supports this notion. For example, studies on entrepreneurs have found a significant role of human capital in explaining entrepreneurs’ network positions, and can mobilize more resources through networks as well (Bhagavatula et al., 2010; Stam, 2010; Methot et al., 2018). In contrast, the utilization perspective, on the other hand, stresses the compensatory or complementary relationships. These studies that examined the utilization of human and social capital show mixed findings. Scholars supporting the compensatory view hold that a simple sum of the two variables cannot guarantee a particular outcome (Johnson et al., 2010), such as positive and negative interaction effects (Brüderl and Preisendörfer, 1998; Klyver and Schenkel, 2013). Others, in light of the complementary view, suggest that, in addition to the joint positive interaction effect, they can also separately result in bringing about a particular outcome (Florin et al., 2003; Batjargal, 2007; Meier et al., 2016).

**FIGURE 1:**
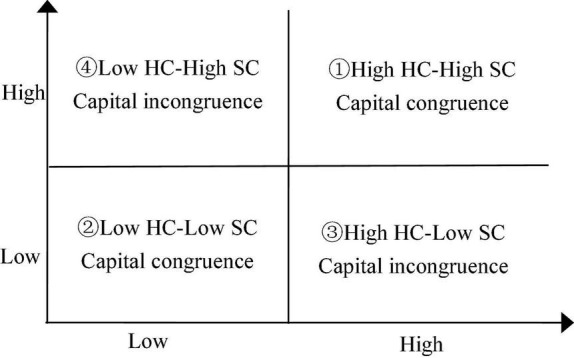
Capital matching situation.

Based on the above-mentioned logical analysis, this study classifies the combination of human capital (HC) and social capital (SC) into four conditions (see Figure 1). To be specific, among the four pairing situations, ① indicates a high level of capital congruence, ② means a low level of capital congruence, while ③, and ④ mean capital in-congruence. The four pairing situations present different synergistic abilities between the two types of capitals.

Recently, research focus has been on emotion management (Amabile et al., 2005; Akgün et al., 2009; Ashkanasy and Humphrey, 2011; Belfanti, 2017). Barsade et al. (2003) summarized the enthusiasm for emotion research in the past 20 years as an *emotional storm*, and claimed that the majority of these studies were conducted at the individual level. An organization, as an aggregation of individuals, has different emotions which are heterogeneous across the workplace (Ashforth and Humphrey, 1995). Hence, much attention is worth being paid to emotional issues at the organizational level. Fineman (1993) extended the construct of emotion to the organizational level and put forward the idea of *organizational emotion*. After the redefinition and improvement of emotional expression in the workplace (Bolton, 2005), research on organizational emotional issues becomes popular in such fields as organizational behavior, and management science and economics (Ashkanasy and Humphrey, 2011). Huy (1999) first developed the concept of dynamic of encouragement, and defined it as a kind of emotional dynamic state and an organizational capability to instill hopes among its members facing radical change efforts.

We, in this study, propose that emotion issue is more likely to be associated with the capital congruence mentioned earlier. According to the resource-based view, the sustainability of competitive advantage hinges on a series of idiosyncratic resources that are difficult to be transferable or replicable (Grant, 1991). Emotional capability is embedded in the interaction of idiosyncratic social networks and knowledge (Huy, 1999). Specifically, when human capital and social capital are congruent, employees with abundant knowledge and social network resources are inclined to put more resources into the process of resource transformation, especially heterogeneous resources, such as emotional resources (Gouldner, 1960). Some scholars further emphasize that emotional dynamism is an internal capability to integrate and reconstruct organizational redundant resources (Huy, 1999; Akgün et al., 2007a,b, 2008, 2009, 2011). Among these redundant resources, human and social capital are the most distinctive and inimitable, and enable firms to rein and convert other organizational resources effectively (Foss and Ishikawa, 2007). However, when human capital and social capital are in-congruent, knowledge, skill, and social network are misaligned in the organizational context, as high ability and low network status, or low ability and high network status, lead to negative emotion among individuals, thereby making it difficult for organizations to instill hopes among all of its members (Huy, 1999; Johnson et al., 2010; Semrau and Hopp, 2016). Based on the theoretical reasoning mentioned above, we posit that the congruence between human and social capital will lead to higher levels of dynamic of encouragement.

***Hypothesis 1a**: Higher congruence between human and social capital is associated with a higher dynamic of encouragement.*

***Hypothesis 1b**: Dynamic of encouragement is higher when human capital is congruent with social capital at high levels rather than at low levels.*

### Human-Social Capital Congruence and Organizational Innovation

According to the resource-based view, an organization is an aggregation of unique resources, and the essence of organizational development is the generation, integration, and reconstruction of those unique resources (Wright and Dunford, 2001). Semrau and Hopp (2016) stated that both human capital and social capital were exclusive organizational resources. More specifically, human capital is the core asset and dominant resource of an organization, which can affect new product development, process improvement, and service upgrading of an organization through dynamic capacity building, generation, interaction, and enabling of different organizational resources (Mohan and Mark, 2005; Engelman et al., 2017). Nahapiet and Ghoshal (1998) took social community as the essence of organizations, and pointed out that social capital, as a network of relationships for resource exchange, coordination, and integration, affected the knowledge value creation of organizations through structure and cognition. Yet, both scholars and practitioners are more and more inclined to believe that different combinations between human and social capitals in entrepreneurial enterprises have differential impacts on specific organizational results, for example, high levels of human and financial social capital could be conducive to entrepreneurial success (Semrau and Hopp, 2016).

Human and social capital have a highly cooperative and matching relationship (Schuller, 2001), while their consequences are diverse under different circumstances (Johnson et al., 2010; Meier et al., 2016). As some scholars suggest, social capital is a kind of relation network of applied knowledge resources, which is tacit and latent, while human capital is more controllable and transferable than social capital (Mosey and Wright, 2007; Stam, 2010). Thus, the synergistic effect of human and social capital is similar to that of explicit and implicit knowledge. Specifically, at the level of *high human capital-high social capital*, organizations win high energy in the capital resources balance, and explicit and tacit knowledge reflecting the inherent consistency in the process of internalization, combination, externalization, and socialization, thereby improving exploitative and exploration innovation of knowledge resource (Nonaka, 1994; Nonaka and Konno, 1998). Drawing on the resource-based view, it is easier for both organizations and employees with more knowledge resources to effectively augment and integrate resource inputs, accelerate the process of resource conversion, and thus transform these resources into innovative intellectual ones (Urgal et al., 2013). By contrast, at the level of *low human capital-low social capital*, although the knowledge resources in the capital are in balance, they are in a state of *low energy* and cannot meet the resource demands in the resource transformation process. Thus, it is difficult to form a fund of resources with potential competitive advantages and innovative enabling ability (Garud and Nayyar, 1994). Based on existing literature, compared with the low level of consistent capital situation, the high level of consistent capital can serve as a buffer and can stimulate synergism between organizational explicit and implicit knowledge, which may consequently drive the enabling process of knowledge resources in the creation and innovation activities. Numerous studies have shown social capital is the connection that combines and disseminates different knowledge or thoughts (Tushman and Anderson, 1986; Snell and Dean, 1992; Zahra and George, 2002; Hill and Rothaermel, 2003). Hargadon and Sutton (1997) considered the combining process as *brokering*, and further proposed that the unconventional combining was vital for innovative resources access. Therefore, it is hypothesized that:

***Hypothesis 2a**: Higher congruence between human and social capital is associated with higher organizational innovation;*

***Hypothesis 2b**: Organizational innovation is higher when human capital is congruent with social capital at high levels rather than at low levels.*

### Dynamic of Encouragement as a Mediator in the Capital Congruence-Organizational Innovation Relationship

We propose that the dynamic of encouragement mediates the linkage between capital congruence and organizational innovation. As mentioned before, the dynamic of encouragement illustrates organizational emotional capability and emotional resources, so it will be more appropriate for us to argue its interactive effects rather than discussing its antecedents or consequences, respectively. On the basis of Sophie and Luc’s (2010) theoretical work, Akhavan and Hosseini (2016) further suggested that capital could affect R&D, which in turn influenced firms’ innovation through shaping organizational capability. To be specific, with dynamic of encouragement, individuals are inspired to bring novel thoughts to old problems, find new problems, and provide innovative solutions (Perel, 2005). On one hand, the hallmarks of capital are network, skill, experience, and so on, while these resources entail an important soft environment provided by emotional integration capabilities to exploit and convert (Huy, 1999; Akgün et al., 2008, 2009, 2011). Previous research has stated that it is critical for knowledgeable employees to break through the technology boundaries of an organization (Kessler et al., 2000) that enhance the emotional capability to deploy and integrate capital resources (Huy, 1999; Akgün et al., 2007b), and further advance the transformation and utilization of prevailing resources, into the innovative resource. On the other hand, Tushman and Anderson (1986) suggest employees with ample skills and social networks are more inclined to alter organizational routines and norms. While inspiring emotions, as a tool of social influence in organizational roles, provide a bridge between a social network and organizational radical change (e.g., routines or prevailing norms) (Hochschild, 1983; Rafaeli and Sutton, 1989; Huy, 1999, 2002; Huy et al., 2014), thereafter shortening the production cycle, updating the organizational process, and altering the management methods. Therefore, it is hypothesized that:

***Hypothesis 3**: The dynamic of encouragement positively mediates the positive relationship between capital congruence and organizational innovation.*

### Environmental Dynamism as a Moderator of the Capital Congruence and Dynamic of Encouragement Relationship

Environmental factors have been regarded as significant boundary conditions known to influence organizational behaviors (Amabile et al., 1996; Dastmalchian, 2010; Delmas and Toffel, 2010). According to the resource dependence theory, an organization is essentially an open system that depends on external resources (Liu et al., 2011; Ganter and Hecker, 2013), and environmental characteristics can influence the chain of “capital-emotion-innovation” (Akgün et al., 2008), particularly environmental dynamics (Jansen et al., 2006). Environmental dynamics means the degree of uncertainty and the pace of change in the environment (Liang and Picken, 2010), in particular toward product and customer demands, raw material supply, and technology (Jansen et al., 2006).

According to Liu et al.’s (2011) work, the process of the organization and management system resources transition is contingent on the organizational environment. Based on the resource-based view, human capital and social capital are concretely presented as knowledge and network resources, respectively. Dynamic of encouragement is regarded as the application of emotional resources, which is consistent with Barrick et al.’s (2015) argument that different forms of resources can be transformed with each other via organizational engagement and motivational antecedents. With different levels of external dynamics, environmental variations will optimize organizational outcomes and promote organizational resources (Garg et al., 2003). On one hand, high environmental dynamism leads to the obsolete of some products, processes, and administration and calls for new ones to replace (Sorensen and Stuart, 2000; Jansen et al., 2005). In this case, the surrounding resources (such as knowledge, skill, and social network) will be mobilized to explore new markets and technologies through integrating emotion among members to solid strategic certainty and skill exploration (Venkatraman, 1989; Teece et al., 1997; Meyer et al., 2009; Ganter and Hecker, 2013), to build gain spiral of organizational resources, and eventually to satisfy the demands of organizational innovative resources (Zahra, 1996). Extant research shows that the perception of hope and encouragement can prompt employees to make use of experience, skill, and social networks to constructively probe inconsistent ideas, diverge from the *status quo*, and motivate risk-taking behaviors (Ludema et al., 1997; Harries, 2003; Akgün et al., 2008). As Jurkiewicz and Giacalone (2004) proposed, when organizations instill hope and happiness among employees, they are more able to handle stress stemming from the work environment, and thus enhance organizational innovation.

On the other hand, in a low uncertainty environment, organizations could foster a climate of satisfaction with the *status quo*, and employees are more likely to save their resources. Therefore, organizational innovation may be hampered due to a loss spiral and innovative resource reduction induced by the lack of consistent values and emotional atmospheres in organizations (Miller and Friesen, 1983). Huy (1999) has argued that for firms facing an increasingly dynamic environment, emotional energy reflects an enormous unexploited resource and enables organizations to realize a strategic stretch. Therefore, it is hypothesized that:

***Hypothesis 4**: Environmental dynamism will moderate the relationship between capital congruence and dynamic of encouragement, thereby improving organizational innovation. High capital congruence is more beneficial for dynamic of encouragement and organizational innovation in a higher dynamic environment.*

**FIGURE 2:**
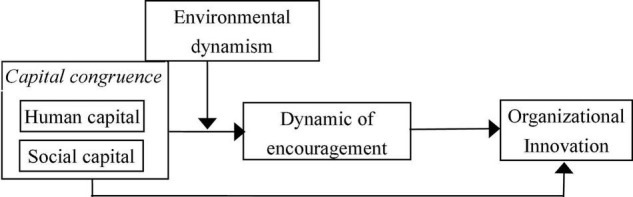
Hypothesized research model.

Hence, we propose a mediated moderation research model to manifest the relationships among all the main variables, as shown in Figure 2.

## Materials and Methods

### Procedures and Samples

To test our hypotheses, we gathered pairing data from R&D teams (employees and their directive leaders) of new ventures in China, including electronic technology, software development, new materials, electronic communications, and machinery manufacturing industries. According to Zahra and Garvis (2000), new ventures were chosen based on the following two standards: (1) the survey was conducted in firms with over 25 employees given that those firms often had formal R&D systems, and (2) we chose firms with ages between 1 and 5 years. To minimize common method deviation, we investigated both employees and their directive leaders. R&D employees were asked to evaluate the dynamic of encouragement, while their leaders reported environmental dynamism, human capital, social capital, and organizational innovation. Participation was voluntary in our survey.

One of the authors is responsible to contact the leaders of target enterprises to communicate about the survey, and we send out the questionnaires to them after their permission. Finally, we delivered 456 questionnaires and 159 were returned during the period from June to September 2020. Of the 159 samples, the distribution of industry was as follows: electronic communication (*N* = 75 firms), software development (*N* = 11 firms), pharmaceuticals (*N* = 7 firms), chemical food (*N* = 31 firms), machinery manufacturing (*N* = 34 firms), and others (*N* = 1 firm). The distribution of organizational age was 1–2 years (54.7%) and 3–5 years (45.3%), while for organizational size was as follows: 25–50 employees (39.6%), 50–200 employees (40.3%), 200–500 employees (5.7%), 500–1,000 employees (13.2%), and over 1,000 employees (1.3%).

### Measurements

To test the hypotheses, we used mature scales from prior studies to measure the constructs in our study. The survey was conducted on a five-point scale, with 1 = never and 5 = always.

#### Human Capital and Social Capital

A 10-item scale proposed by Subramaniam and Youndt (2005) was used to assess the human capital and social capital, with five items for human capital and the other five items for social capital. All items presented the holistic capability to share and manage knowledge among employees. One sample item was “our employees are skilled at collaborating with each other to diagnose and solve problems.” The internal consistency reliability for human capital and social capital were 0.899 and 0.876, respectively.

#### Dynamic of Encouragement

We used a three-item scale developed by Akgün et al. (2007a,2009) to measure the dynamic of encouragement. One sample item was “our firm has ability to facilitate the variety of authentic emotions that legitimately can be displayed.” The internal consistency reliability for it was 0.850.

#### Environmental Dynamism

We measured environmental dynamism in terms of instability and the changing pace of the external environment. Each aspect was assessed with a three-item scale proposed by Jansen et al. (2006). The internal consistency reliability of environmental dynamism was 0.770.

#### Innovation Performance

We used a nine-item scale from Jiménez-Jiménez and Sanz-Valle (2008) to measure innovation performance. To be specific, three items were used for product innovation; three items for process innovation; and the remaining three items for administrative innovation. It was also checked on a five-point scale (1 = below competitors, 3 = similar competitors, and 5 = above competitors). The internal consistency reliability for innovation performance was 0.729.

#### Control Variables

Following previous studies (Akgün et al., 2007b,^2008^, ^2009^, ^2011^), we controlled organizational type, age, and size in this study, considering organizational characteristics were found to influence organizational performance and emotional capability.

### Data Analysis

We conducted the confirmatory factor analysis (*CFA*) on all variables to evaluate the validity (Table 1) using Lisrel8.7. The results of CFA suggest that factor loadings are all above 0.55, AVE above 0.5, and CR above 0.8, which are acceptable.

**TABLE 1:** Confirmatory factor analysis and scale reliability.

Variables	Indicates	Factor loading	CR	AVE
Human capital (α = 0.899)	HC1	0.875	0.906	0.660
	HC2	0.704		
	HC3	0.841		
	HC4	0.830		
	HC5	0.801		
Social capital (α = 0.876)	SC1	0.739	0.908	0.664
	SC2	0.794		
	SC3	0.851		
	SC4	0.809		
	SC5	0.874		
Dynamic of encouragement (α = 0.850)	DOE1	0.839	0.896	0.741
	DOE2	0.866		
	DOE3	0.877		
Environmental dynamism (α = 0.770)	ED1	0.809	0.886	0.722
	ED2	0.860		
	ED3	0.879		
Organizational innovation (α = 0.729)	OI1	0.824	0.926	0.585
	OI2	0.797		
	OI3	0.619		
	OI4	0.735		
	OI5	0.846		
	OI6	0.787		
	OI7	0.793		
	OI8	0.748		
	OI9	0.707		

Table 2 presents the descriptive statistics and correlations among the variables. As expected, all independent variables were significantly associated with dynamic of encouragement (*r* = 516, *p* < 0.01; *r* = 471, *p* < 0.01) and organizational innovation (*r* = 484, *p* < 0.01; *r* = 402, *p* < 0.01). This finding provides initial support for the hypotheses test. In addition, all the correlations of the variables were inferior to their corresponding square roots of AVE.

**TABLE 2:** Mean, standard deviation, and correlation.

Variables	M	SD	1	2	3	4	5	6	7	8
*1. Age*	1.450	0.499	N							
*2. Size*	3.960	2.153	0.001	N						
*3. Type*	1.960	1.049	0.420***	−0.239***	N					
*4. HC*	3.416	0.580	−0.581***	−0.241***	−0.399***	(0.848)				
*5. SC*	3.491	0.556	−0.345***	−0.416***	−0.307***	0.781***	(0.819)			
*6. DOE*	3.476	0.548	−0.153*	0.222***	−0.560***	0.516***	0.471***	(0.885)		
*7. ED*	3.390	0.747	0.493***	–0.085	0.479***	0.558***	0.532***	0.186**	(0.856)	
*8. IP*	3.442	0.327	−0.291***	0.031	−0.349***	0.484***	0.402***	0.394***	0.323***	(0.660)

*M, mean; SD, Standard deviation.*

*P < 0.1; **P < 0.05; ***P < 0.01; N = 159.

*Age:1 = 1–2 years, 2 = 3–5 years; Size:1 = 25–50 employees, 2 = 50–200 employees, 3 = 200–500 employees, 4 = 500–1,000 employees, 5 = more than 1,000 employees; Type:1 = electronic communication, 2 = machinery manufacturing, 3 = pharmaceuticals, 4 = chemical food, 5 = software development, 6 = others; HC, Human capital; SC, Social capital; DOE, Dynamic of encouragement; ED, Environmental dynamism; IP = Organizational innovation; the square root of AVE is shown in parentheses along the diagonal.*

#### Test of Hypotheses

Polynomial regression with response surface methodology was used to test our hypotheses. Response surface methodology is fit to investigate the extent to which two predictors, namely component measures, and their mutual consistency (congruence) and discrepancy (in-congruence) associate with an outcome variable (Edwards and Parry, 1993; Shanock and Heggestad, 2010). In our study, the outcome variable is the dynamic of encouragement or organizational innovation, and the two component measures are human capital and social capital. The equation of polynomial regression is *Z* = *b_0_* + *b_1_X* + *b_2_Y* + *b_3_X^2^* + *b_4_X* × *Y* + *b_5_Y^2^* + *e*, where Z is the dynamic of encouragement or organizational innovation, X is the human capital, and Y is the social capital. We then plotted the three-dimensional response surfaces through polynomial regression where HC (Human capital) and SC (Social capital) were scaled on the perpendicular horizontal axes, while DOE or IP was plotted on the vertical axis. The results are outlined in Table 3.

**TABLE 3:** The results of polynomial regression.

Variable	DOE				IP			
		
	M1		M2		M3		M4	
				
	*B*	*se*	*B*	*se*	*B*	*se*	*B*	*se*
*Constant*	3.841***	0.137	1.111***	0.373	3.803***	0.092	2.338***	0.299
*Age*	0.098	0.080	0.313***	0.074	−0.112**	0.054	0.118**	0.059
*Size*	0.021	0.017	0.100***	0.015	–0.006	0.012	0.013	0.012
*Type*	−0.302***	0.039	−0.215***	0.035	−0.089***	0.026	–0.040	0.028
*HC(b_1_)*			0.106	0.113			0.427***	0.090
*SC(b_2_)*			0.470***	0.104			–0.135	0.083
*HC Squared(b_3_)*			−0.331***	0.067			0.055	0.054
*HC* × *SC (b_4_)*			0.439***	0.107			0.093	0.086
*SC Squared(b_5_)*			–0.079	0.055			−0.154***	0.044
*R* ^2^	0.328		0.652		0.148		0.373	
*HC* = *SC congruence line*								
*Slope (b1* + *b2)*			0.576**				0.292**	
*Curvature (b3* + *b4* + *b5)*			0.029*				–0.006	
*HC* = *-SC in-congruence line*								
*Slope (b1–b2)*			–0.364**				0.562**	
*Curvature (b3–b4* + *b5)*			–0.849***				–0.192*	

*P < 0.1; **P < 0.05; ***P < 0.01.

Hypothesis 1a proposes that high congruence between human and social capital is associated with a high dynamic of encouragement. Model 2 reported that the curvature along the in-congruence line (b3–b4 + b5) is negative and significant (−0.849, *p* < 0.05), indicating that the surface along the in-congruence line is convex. In addition, the slope (b1–b2) of the surface along the in-congruence line is also significant (−0.364, *p* < 0.05), which suggests that the dynamic of encouragement reaches maximization along the in-congruence line at the point of congruence. This means the ridge of the surface runs along the congruence line. Thus, Hypothesis 1a is supported.

Hypothesis 1b states that the dynamic of encouragement is maximized when human and social capital are congruent. To test H1b, we check the shape (b1 + b2 measuring slope and b3 + b4 + b5 measuring curvature) of the curve along the congruence line. According to Model 2, the slope of the line of capital congruence line (HC = SC) associated with DOE is 0.576 (*p* < 0.05), and the curvature along the line of capital congruence associated with DOE is 0.029 (*p <* 0.1). This suggests the surface is non-linear (curved) essentially, and the slope of the surface increases over the congruence line. Hypothesis 1b is supported.

In a similar vein, Model 4 presented that the curvature along the in-congruence line (b3–b4 + b5) is also significant yet negative (−0.192, *p* < 0.1), suggesting that organizational innovation is stronger at high HC-low SC combination rather than low HC-high SC combination. Yet, the positive and significant slope (b1–b2) of the surface along the in-congruence line (0.562, *p* < 0.05) suggests that organizational innovation will be maximized along the in-congruence line at the point of congruence. Meanwhile, the slope of the line of capital congruence (b1 + b2) is significant (0.292, *p* < 0.05), and the resulting slope of the surface indicates that organizational innovation is stronger at high HC-high SC than low HC-low SC combination. The curvature along the congruence line (b3 + b4 + b5) is not significant (–0.006, ns.), meaning the surface has a plane ridge operating along the line of congruence. Hypothesis 2, which argues that high congruence between human and social capital will lead to better organizational innovation, is thus supported.

**FIGURE 3:**
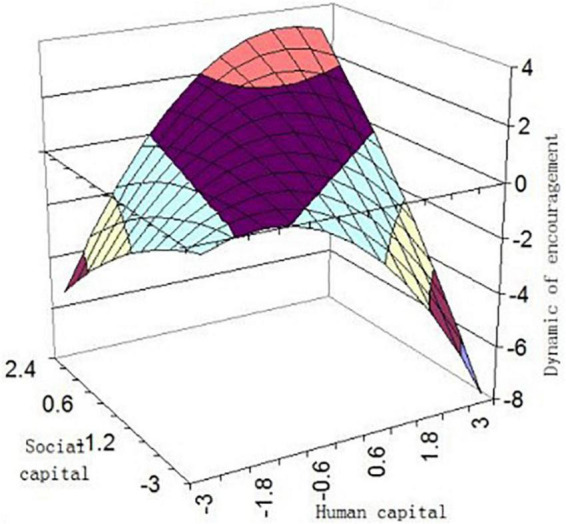
Dynamic of encouragement as predicted from capital.

**FIGURE 4:**
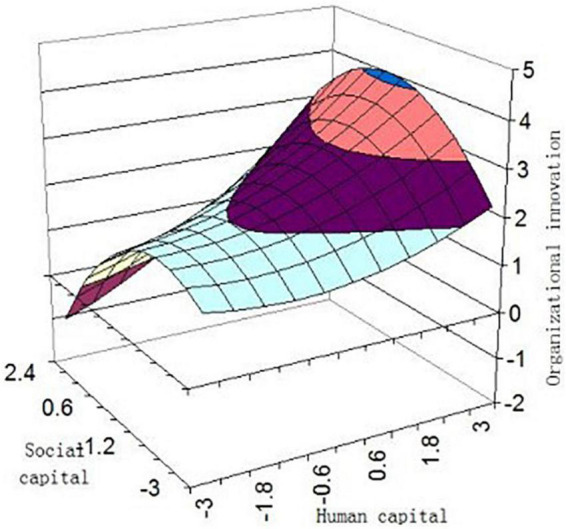
Organizational innovation as predicted from capital.

We depicted a three-dimensional response surface to further interpret our findings (Figures 3, 4). The graph shows DOE (IP) will be higher when HC and SC are consistent, such that DOE (IP) reaches its highest level in high HC-high SC combination, and DOE (IP) was higher in low HC-low SC than in the other two incongruent combinations (high HC-low SC or low HC-high SC). Hypotheses 1 and 2 were further verified.

We used mixed regression models to check the hypotheses through separate steps. Hypothesis 3 claims that the dynamic of encouragement positively mediates the positive linkage of capital congruence to organizational innovation. Table 4 reports the results of mediation effects through bootstrapping. Following Edwards’ (2002) work, we used 5,000 bootstrap samples to build a percentile-based 95% CI (95-percent confidence interval) around the mediation effects. The resulting intervals of indirect and total effects are found to be [0.020, CI (0.005, 0.042)] and [0.043, CI (0.010, 0.077)], which excludes zero. The result of direct effect in 95% bias-corrected confidence interval [0.023, CI (–0.013, 0.059)] includes zero, suggesting that the dynamic of encouragement absolutely mediates the relationship between positive capital congruence and organizational innovation. In addition, we used the Sobel test to check the indirect effects to further verify the mediation effects. The Sobel test value of HC × SC was 2.517 (*p* < 0.05), further supporting Hypothesis 3.

**TABLE 4:** Bootstrapping of mediation effects.

Outcome variable	Independent variable	Sobel test	Direct and indirect	Effects	se.	95% CI	
	
						LLCI	ULCI
*IP*	*HC* × *SC*	2.517**	*Indirect effect*	0.020	0.009	0.005	0.042
			*Direct effect*	0.023	0.018	−0.013	0.059
			*Total effect*	0.043	0.017	0.010	0.077

**P < 0.05.

Hypothesis 4 proposes that environmental dynamism moderates the relationship between capital congruence and dynamic of encouragement, and high capital congruence is more beneficial for dynamic of encouragement in a highly dynamic environment. Referring to Hayes (2013), we utilized the bootstrapping method (95% confidence interval, sample = 5,000) to test the moderated mediation, and the results are displayed in Table 5. High ED and low ED were one standard deviation above and below the mean, respectively. The results suggested that capital congruence (*HC* × *SC*) and DOE had a significant and positive impact on IP (β = 0.042, CI (0.008, 0.087)] for the high ED group. Hypothesis 4 was thus supported.

**TABLE 5:** Bootstrapping of moderated mediation.

Mediator variable	Independent variable	Conditional indirect effect					Index of moderated mediation			
		
		Moderator variable	Effects	se.	95% CI		INDEX	se.	95% CI	
								
					LLCI	ULCI			LLCI	ULCI
*DOE*	*HC* × *SC*	*High ED*	0.054	0.024	0.009	0.103	0.042	0.020	0.008	0.087
		*Low ED*	−0.007	0.010	−0.033	0.008				

**FIGURE 5:**
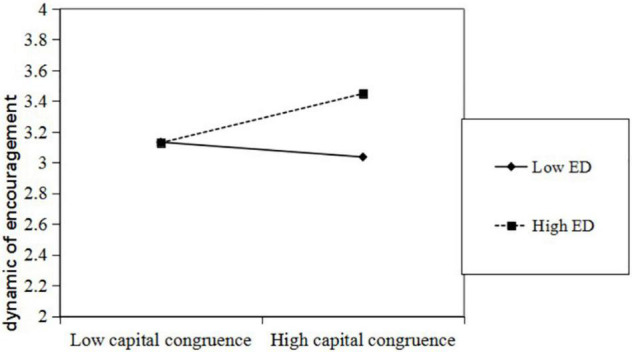
Effects of the interaction between capital congruence and environmental dynamism on dynamic of encouragement.

To interpret the moderating effect, we regressed equations on the linkage of capital congruence and dynamic of encouragement at the high and low levels of environmental dynamism. Referring to Cohen and Cohen’s (1983) work, the high and low values were defined as one standard deviation above and below the mean value of environmental dynamism. According to Figure 5, the plots of the interactions present that capital congruence is related to the dynamism of encouragement under high environmental dynamism. In contrast, the flat slope shows that capital congruence cannot significantly affect the dynamism of encouragement under low environmental dynamism. Thus, Hypothesis 4 was supported.

## Discussion

The present study advances our insights into how different types of capital affect innovation by focusing on the congruence between human capital and social capital. Drawing on the resource-based theory, we argue that the congruence of human and social capital serves as an important resource for organizational innovation. Furthermore, the dynamic of encouragement fully mediated the influence of capital congruence on innovation. In addition, the influence of capital congruence on organizational innovation was contingent upon the environmental dynamism. Data from over 200 technological new ventures support all our hypotheses.

### Theoretical Implications

Our findings make several theoretical contributions to the literature on organizational behavior and human resource management. First, recent research shows a debatable result regarding the interactive effectiveness of human and social capital, namely, compensatory relationship (Brüderl and Preisendörfer, 1998; Klyver and Schenkel, 2013) and complementary relationship (Coleman, 1988; Florin et al., 2003; Klyver and Schenkel, 2013). Based on the resource-based theory, our study suggests neither fundamentally complementary nor fully compensatory claims between human capital and social capital (Semrau and Hopp, 2016). The findings confirm the conclusion that both human capital and social capital are critical organizational resources, and that all kinds of resources are converted to each other in an efficient or effective manner, as proposed by Miller et al. (2015). Thus, our study, on one hand, reconciles previous contradicting theoretical claims (compensatory or complementary) and offers direct evidence for a significant relationship between human capital and social capital in dynamic environments on the other.

Second, the findings of our study illustrate the mediation role of dynamic of encouragement in the linkage between human and social capital and organizational innovation performance. Innovation has been considered as a primary contributor to the development and survival of new ventures (Vera et al., 2016). Our study suggests that when a firm can instill hope and enthusiasm among its members, it is more likely to make full use of the knowledge and social network resources: (1) To constantly renovate its manufacturing process, improve its business process, and thus alter its production approach; and (2) to duly launch novel products and services.

Third, our work contributes to capital development and organizational capability by exploring their relationship in a complex context. Our results indicate that in a highly dynamic environment, capital congruence can be more beneficial for dynamic of encouragement, thereafter improving organizational innovation. In addition, a low dynamic environment cannot significantly influence the relationship between capital congruence and the dynamic of encouragement. This finding is not surprising, as new ventures are typical organizations with a strong innovative inclination and resource demands. Yet, the unique characteristics that new ventures show in the creative and innovative activities, for example, path ambiguity, time limitations, and skill specificity, suggest that new ventures are more sensitive to the dynamic environment.

### Managerial Implications

Our study also provides some managerial implications for new ventures to boost organizational capability and innovation performance. First, managers should realize the importance of employees’ emotions, and further utilize employees’ emotions in both the innovation process and structure of the organizational daily work. Specifically, managers ought to rein employees “emotions, arouse employees” enthusiasm, and inspire them to achieve their goals. For example, managers could create an atmosphere of open dialog and interactions for employees through after-work meetings, to reconcile employees’ emotional divergence. Second, start-ups should take advantage of their innovative capability to gain and maintain a competitive advantage. For new ventures, innovations are primarily rooted in social network construction and knowledge application. Thus, new ventures should pay more attention to resource involvement, particularly the significance of knowledge and social network resources, and realize the role of emotional dynamics in the conversion of different resources under different environmental conditions.

### Limitations

Some limitations in this study need to be addressed in future studies. First, since the data were collected through a multi-source survey, we adopted some statistical methods (Podsakoff and Organ, 1986) to examine common method variance. Yet, despite this, we cannot rule out the bias due to subjectivity. Future studies could use different assessors to measure the main variables and gather data through multiple time periods. Second, we merely focused on high-tech start-ups. As a result, it remains unclear whether our findings can be applied to other industries. Future studies could use samples from other industries to verify the conclusions of our study.

## Conclusion

Organizations must overcome environmental dynamism to seek further development, and innovation is regarded as a crucial factor to manage environmental dynamics. In this study, we propose a moderated mediation model to manifest the relationships among capital (human capital and social capital) congruence, dynamic of encouragement, environmental dynamism, and organizational innovation. Using data from more than 200 technological new ventures in China, we find that organizational innovation is stronger when human and social capital are congruent, the dynamic of encouragement fully mediates the relationship between capital congruence and organizational innovation performance, and environmental dynamism positively moderates the relationship between capital congruence and dynamic of the environment, such that the relationship is stronger for new ventures in higher rather than lower dynamic environments.

## Data Availability Statement

The raw data supporting the conclusions of this article will be made available by the authors, without undue reservation.

## Ethics Statement

The authors declare ethical review and approval was not required for the study on human participants in accordance with the local legislation and institutional requirements. All participants received the questionnaires in an envelope with an introduction of the study purposes as well as a written informed consent form. All participants provided written informed consent, affirmed their understandings of the study purposes and that they would like to participate in this study voluntarily.

## Author Contributions

YL contributed to data collection, writing, and editing. XB participated in data collection and collation. WC handled interpretation and contributed to revision. All authors contributed to the article and approved the submitted version.

## Conflict of Interest

The authors declare that the research was conducted in the absence of any commercial or financial relationships that could be construed as a potential conflict of interest.

## Publisher’s Note

All claims expressed in this article are solely those of the authors and do not necessarily represent those of their affiliated organizations, or those of the publisher, the editors and the reviewers. Any product that may be evaluated in this article, or claim that may be made by its manufacturer, is not guaranteed or endorsed by the publisher.
